# Welcome to *Journal of Foot and Ankle Research*: a new open access journal for foot health professionals

**DOI:** 10.1186/1757-1146-1-1

**Published:** 2008-07-28

**Authors:** Hylton B Menz, Mike J Potter, Alan M Borthwick, Karl B Landorf

**Affiliations:** 1 Musculoskeletal Research Centre, Faculty of Health Sciences, La Trobe University, Bundoora, Victoria, Australia; 2 School of Health Sciences, University of Southampton, Southampton, UK; 3 Department of Podiatry, Faculty of Health Sciences, La Trobe University, Bundoora, Victoria, Australia

## Abstract

*Journal of Foot and Ankle Research *(*JFAR*) is a new, open access, peer-reviewed online journal that encompasses all aspects of policy, organisation, delivery and clinical practice related to the assessment, diagnosis, prevention and management of foot and ankle disorders. *JFAR *will cover a wide range of clinical subject areas, including diabetology, paediatrics, sports medicine, gerontology and geriatrics, foot surgery, physical therapy, dermatology, wound management, radiology, biomechanics and bioengineering, orthotics and prosthetics, as well the broad areas of epidemiology, policy, organisation and delivery of services related to foot and ankle care. The journal encourages submission from all health professionals who manage lower limb conditions, including podiatrists, nurses, physical therapists and physiotherapists, orthopaedists, manual therapists, medical specialists and general medical practitioners, as well as health service researchers concerned with foot and ankle care. All manuscripts will undergo open peer review, and all accepted manuscripts will be freely available on-line using the open access platform of BioMed Central.

## Background

*Journal of Foot and Ankle Research *(*JFAR*) is a new, open access peer-reviewed online journal that encompasses all aspects of policy, organisation, delivery and clinical practice related to the assessment, diagnosis, prevention and management of foot and ankle disorders. *JFAR *is the official research publication of the Society of Chiropodists and Podiatrists (UK) and the Australasian Podiatry Council. However, the Editorial Board encourages submission from all health professionals who manage lower limb conditions, including podiatrists, nurses, physical therapists and physiotherapists, orthopaedists, manual therapists, medical specialists and general medical practitioners, as well as health service researchers concerned with foot and ankle care.

**Figure 1:**
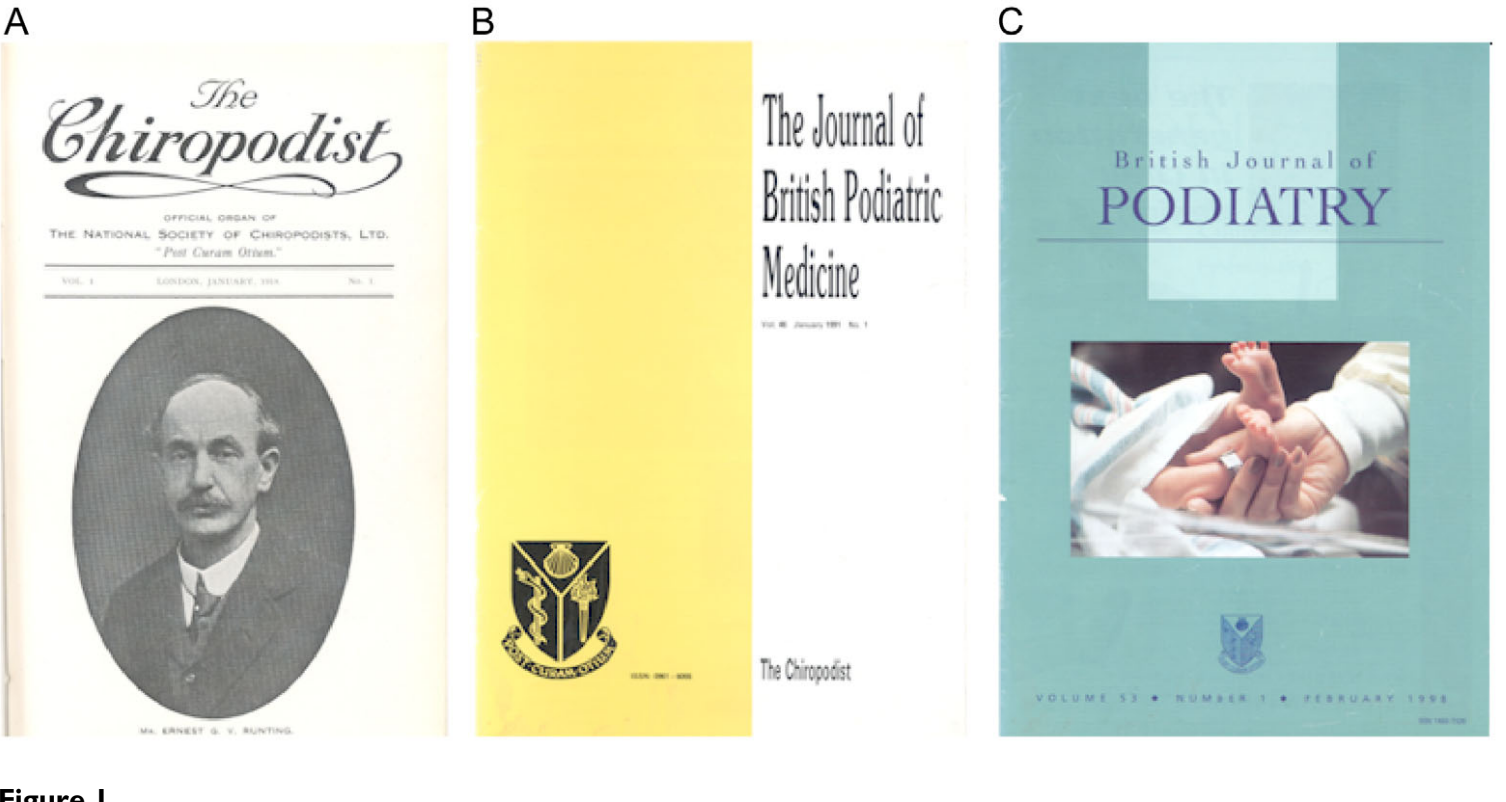
**Chronology of chiropody/podiatry publications in the UK**. A: *The Chiropodist*, B: the *Journal of British Podiatric Medicine*, C: the *British Journal of Podiatry*.

*JFAR *builds upon a long and proud history of scholarly publication in the podiatry profession. The first foot-related journal in the UK, *The Chiropodist*, was published by the National Society of Chiropodists in January 1914. The National Society of Chiropodists was wound up in 1915, to be replaced with the Incorporated Society of Chiropodists, which published its first journal as a continuation of *The Chiropodist*. Following the amalgamation of the Incorporated Society of Chiropodists with four other bodies at the end of 1945, the newly formed Society of Chiropodists produced its first journal, still called *The Chiropodist*, in January 1946. This incarnation of the journal was continuous up until January 1989, when it was merged with the *British Journal of Chiropody *(originally the journal of the British Association of Chiropodists, later becoming an independent outlet after the Association merged with the Society of Chiropodists in 1945). The new journal was still called *The Chiropodist*, but with a new sub-title: *The Journal of British Podiatric Medicine*, which became the full title in 1991. The final manifestation of the journal – the *British Journal of Podiatry *– represented a merger between *The Journal of British Podiatric Medicine *and the *British Journal of Podiatric Medicine and Surgery*, which occurred in response to the amalgamation of the Podiatry Association, the Association of Chief Chiropody Officers and the Society of Chiropodists to form the Society of Chiropodists and Podiatrists in 1998 [1]. Cover images of the three main phases of the UK journal are shown in Figure 1.

**Figure 2:**
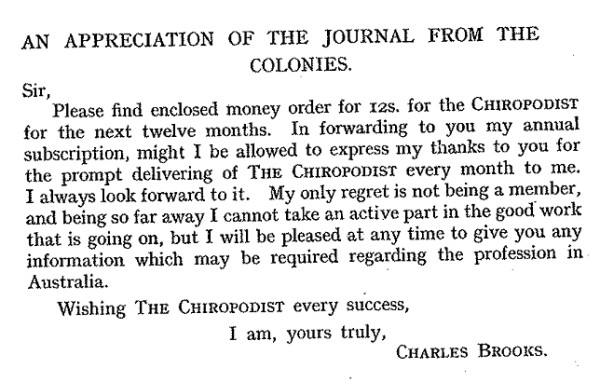
**Letter to the editor of *The Chiropodist *(1924, Vol. 11, p 225) from a satisfied Australian subscriber**.

**Figure 3:**
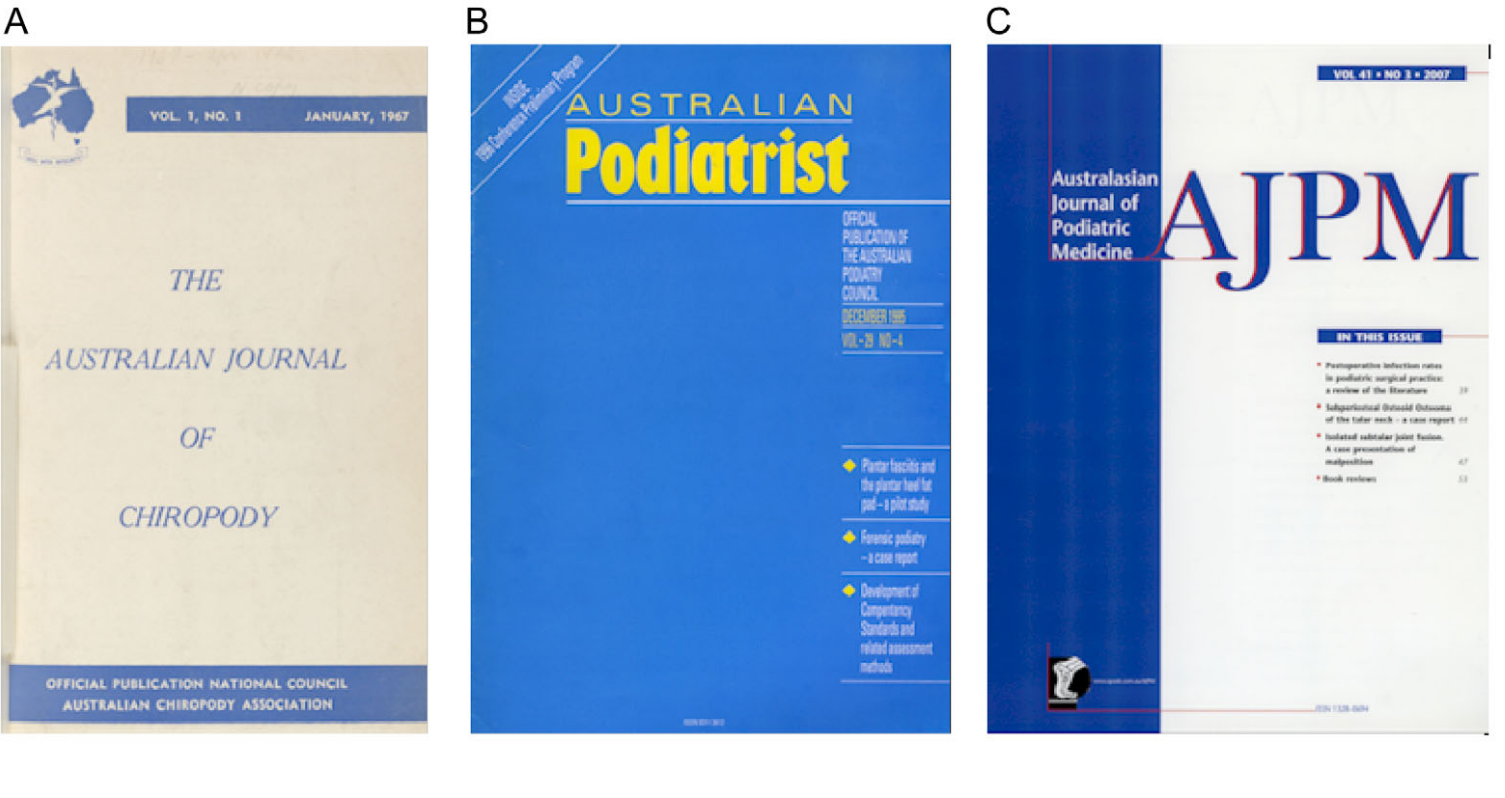
**Chronology of chiropody/podiatry publications in Australia**. A: *The Australian Journal of Chiropody*, B: *Australian Podiatrist*, C: the *Australasian Journal of Podiatric Medicine*.

The chiropody/podiatry profession in Australia and New Zealand was strongly influenced by the UK model, and despite considerable delays in the dispatch processes of the journal, it would appear that *The Chiropodist *did have a small number of eager subscribers in "The Colonies" (Figure 2). However, it was not until 1966 that the first podiatry journal in Australia (the *Australian Journal of Chiropody*) was published. This journal was renamed the *Australian Podiatrist *in 1974. Most recently, following the amalgamation of the Australian Podiatry Council and the New Zealand Society of Podiatrists to form the Australasian Podiatry Council in 1997, the *Australian Podiatrist *merged with the *New Zealand Journal of Podiatric Medicine *to form the *Australasian Journal of Podiatric Medicine *(see Figure 3).

The late 1990s witnessed an ongoing debate regarding the future of scholarly publishing in the Australasian and UK podiatry professions which arose from two related issues: the growing need for podiatry researchers to publish in high profile journals, and the drive towards internationalisation [2-5]. During this period, it was clear that sustaining the two 'local' journals as credible research publications was becoming increasingly difficult [6], as podiatry researchers were turning to more prestigious, higher profile journals indexed by Medline [7,8]. Indeed, a survey of podiatry academic staff in Australia revealed that the *British Journal of Podiatry *and the *Australasian Journal of Podiatric Medicine *were considered to be the least prestigious of all foot and ankle publications, and that academic staff considered inclusion in Medline to be the most important factor to consider when selecting a journal to publish in [7].

**Figure 4:**
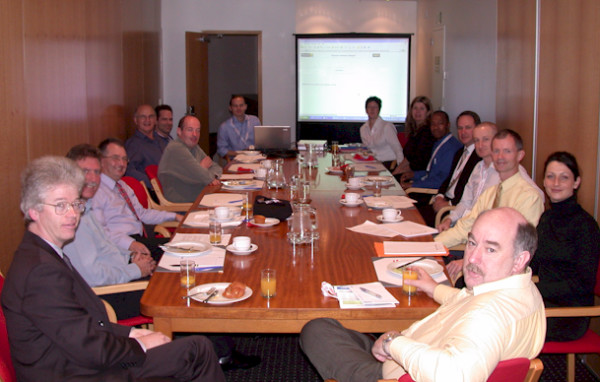
**Breakfast meeting to discuss the new journal at the 21^st ^Australasian Podiatry Conference, Christchurch, New Zealand, 3.9.2005**. From left to right: Wayne Tucker, Stuart Baird, Mike Potter, Prof Keith Rome, John Price, Matthew Dilnot, A/Prof Hylton Menz, Alison Petchell, Carol Mioduchowski, Richard Masoetsa, Matthew Slattery, Dr Karl Landorf, Dr Alan Borthwick, Dr Anita Raspovic and Craig Payne.

In 2005, tentative steps were taken to extend the degree of collaboration between the two organisation's journals via the reciprocal publication of selected papers (so-called 'international papers'). Finally, following several discussions between 2005 and 2006, including a meeting held during the 21^st ^Australasian Podiatry Conference in Christchurch (see Figure 4), the Australasian Podiatry Council and Society of Chiropodists and Podiatrists reached an agreement to develop a new journal. Subsequently, the final edition of the *British Journal of Podiatry *(Vol. 10, No. 4) was published in November 2007 [9], and the final edition of the *Australasian Journal of Podiatric Medicine *(Vol. 41, No. 3) was published in December 2007 [10], paving the way for the development of the new journal, *Journal of Foot and Ankle Research (JFAR)*.

## Why open access publishing?

The re-evaluation of scholarly publishing by the Australasian Podiatry Council and Society of Chiropodists and Podiatrists coincided with a major upheaval in biomedical publishing. Motivated by the growth of the internet and a desire to enable wider access to scientific information, the concept of open access publishing gathered considerable momentum in the late 1990s [11-14]. Open access publishing enables researchers to submit manuscripts to web-based journals that can be downloaded free of charge by anyone with an internet connection, with no subscription or registration barriers. The costs of publishing are borne by the author, or, in most cases, by the author's institution or funding body. In this way, access to scholarly information is vastly increased, and the costs of publishing research are contained. BioMed Central [15], the publisher of *JFAR*, is the world's largest open access publishing company, and currently produces 186 journals.

Open access publishing with Biomed Central offers several significant benefits over the traditional publication model [16], including:

(i) Rapid peer review: the peer review process in many journals is often frustrating and time-consuming, and may in some cases take over 12 months to complete. For *JFAR*, this process is streamlined due to BioMed Central's web-based system for submission and for referees to view manuscripts and return their reviews.

(ii) High visibility and accessibility: publishing in *JFAR *provides authors with access to a truly global readership in medicine and allied health. PubMed [17], the world's most popular literature search service, indexes all research published in BioMed Central journals. It is worth noting that the precursor journals to *JFAR *– the *Australasian Journal of Podiatric Medicine *and the *British Journal of Podiatry *– were not indexed in PubMed, and several current foot and ankle journals, such as *Foot and Ankle Surgery *and *The Foot *are also not indexed in PubMed. BioMed Central also participates in CrossRef [18] and the Open Citation Project [19], allowing direct linking from citations to the full text of the article in a BioMed Central journal.

(iii) Vastly reduced time period between acceptance of a manuscript and its publication: for most journals, it can take between six and 24 months from acceptance of a paper to final availability in hard copy. This delay occurs because of the need to produce paper issues on a monthly, bimonthly or quarterly basis with a fixed number of pages, which can create a substantial publishing backlog. With *JFAR*, this unnecessary delay is removed, and articles will be published on-line within a few days of editorial acceptance.

(iv) The author retains copyright: with traditional journals, the author transfers ownership of their article to the publisher, who can legally restrict access to the article (even to the point of restricting sharing of the article between colleagues). With open access journals, the author retains copyright and grant any third party the right to use the article freely, as long as its integrity is maintained and its original authors, citation details and publisher are identified.

(v) Web-based flexibility: traditional print journals are forced to restrict the size of articles due to the cost of producing printed issues. In some journals, articles are restricted to 2,000 words or less, which creates difficulties for authors and may require the deletion of potentially important information. In addition, many journals charge the author for reproduction of colour images. In contrast, the web interface of BioMed Central journals allows for larger articles, and the unrestricted inclusion of non-written material such as high resolution colour photographs, additional datasets and movie files.

Articles published in open access journals are subject to an article processing charge, which helps fund the journal and enables free access to articles worldwide. If the submitting author of a manuscript belongs to an institution that has a subscription to BioMed Central, the article processing charge is waived. There are currently 319 Members and Supporter Members in 34 countries [20]. Authors who do not belong to a BioMed Central member institution pay an article processing charge upon publication. However, as part of the agreement formulated between the Australasian Podiatry Council and the Society of Chiropodists and Podiatrists, all Association/Society members who wish to submit a manuscript will have their article processing charge covered.

## What is the role of *JFAR*?

Scholarly journals exist primarily as an avenue for the dissemination of research findings. For clinical journals, this dissemination should ideally encompass both "researcher to researcher" and "researcher to clinician" transfer of information, in order to educate clinicians and ultimately improve clinical outcomes for patients. However, it has been argued that journals have several broader roles, such as promoting and reforming the professions they serve [21]. As *JFAR *is supported by the premier bodies of the podiatry professions in Australasia and the UK, the journal will inevitably have a strong podiatry emphasis. However, the editors acknowledge that the management of foot conditions encompasses a range of health care professionals [22], and that the structure, education and scope of practice of these professions varies considerably between different countries. *JFAR *will therefore welcome all manuscripts that advance our understanding of foot disorders, irrespective of the professional background or country of origin of the contributing authors. In doing so, we hope to foster greater awareness and collaboration between foot health professionals, and help break down historical, national and political barriers impeding the progress and advancement of foot and ankle care.

## What will be published in *JFAR *and how will it be reviewed?

*JFAR *will focus primarily on original research articles, but will also publish editorials, methodology articles, letters to the editor, study protocols and reviews. Case reports will only be considered if they provide unique or important additional insights into the causes or treatment of foot and ankle disorders. In addition, case reports must be evidence-based where good evidence is available [23]. Manuscripts submitted to *JFAR *will initially be reviewed by the editors and subsequently by two external peer reviewers. Reviewers will be asked to indicate whether the manuscript is scientifically sound, relevant and also to indicate the level of interest to foot health professionals.

*JFAR *operates a fully open peer review system, meaning that the identity of the authors is known to the reviewers, and vice versa. There is no evidence that such a system produces better quality reviews or changes reviewers' recommendations compared to traditional "closed" peer review [24]. However, open peer review is a far more transparent system, potentially fosters greater accountability on the part of reviewers, and may also prevent potentially serious abuses of the system (such as reviewers stealing authors' ideas or intentionally slowing the progress of a competitor's manuscript) [21]. All correspondence pertaining to the peer review process, including peer reviewers' comments, authors' replies and revisions of the manuscript will be freely accessible from the 'prepublication history' section of each article published on the *JFAR *website.

Within a few days after acceptance in *JFAR*, provisional versions of articles will be published on our website [25] as Portable Document Files (PDFs). The provisional version corresponds to the manuscript as it appeared upon final acceptance by the editors, and the reference will appear in PubMed as an 'Epub ahead of print'. Fully formatted PDF and full text (HTML) versions with cross-linked reference lists are made available shortly afterwards. This system allows the full text of research atricles to be rapidly accessible to clinicians and researchers and vastly reduces the turnaround time between submission and publication. Final versions will be accessible and searchable through the *JFAR *web archives, as well as PubMed, Google Scholar, Scopus and several other scholarly databases. In addition to providing much wider dissemination of research articles (BioMed Central articles have an average of 2,000 downloads in the first 12 months of publication [26]), this approach also benefits authors, as open access papers have been shown to attract more citations than non-open access articles [27].

## Where to from here?

The editors of *JFAR *hope that the journal will be entertaining, educational, provocative and clinically useful to all health care professionals involved in the management of foot and ankle disorders. We look forward to receiving your submissions, general feedback as to how the journal is progressing and suggestions for how it can be improved.

## Authors' contributions

All authors assisted with drafting the manuscript, and all authors read and approved the final manuscript.
